# Porta Hepatis Paraganglioma: A Rare Case Report With Diagnostic Pitfalls

**DOI:** 10.1155/crip/4237665

**Published:** 2026-10-21

**Authors:** Maryam Vajihinejad, Soroosh Najafi, Amirpasha Amlelshahbaz, Sayed Ahmadreza Saeed

**Affiliations:** ^1^ Department of Pathology Shahid Sadoughi University of Medical Sciences Yazd Iran ssu.ac.ir; ^2^ School of Medicine Shahid Sadoughi University of Medical Sciences Yazd Iran ssu.ac.ir; ^3^ Department of Radiology Shahid Sadoughi University of Medical Sciences Yazd Iran ssu.ac.ir

**Keywords:** case report, diagnostic errors, large-core needle biopsy, paraganglioma, porta hepatis

## Abstract

Paragangliomas are rare neuroendocrine tumors that infrequently localize to the porta hepatis, where they create significant diagnostic challenges by mimicking hepatocellular carcinoma or distinct mesenchymal neoplasms. We report the case of a 64‐year‐old woman presenting with vague abdominal pain and an enhancing porta hepatis mass compressing the inferior vena cava. An initial core needle biopsy, which suggested a benign mesenchymal tumor, underscores the potential for misdiagnosis in such atypical presentations. During surgery, the patient developed paroxysmal hypertension suggestive of occult catecholamine release. Subsequent histopathological examination revealed a classic zellballen pattern, with immunohistochemical positivity for Chromogranin A, Synaptophysin, and S100 definitively confirming a paraganglioma. The patient remains disease‐free at a 2‐year follow‐up. This case highlights the importance of considering paraganglioma in the differential diagnosis of porta hepatis masses and emphasizes the value of histopathology and immunohistochemistry for accurate diagnosis, particularly in patients who lack preoperative adrenergic symptoms.

## 1. Introduction

Pheochromocytomas and paragangliomas, collectively referred to as PPGLs, are catecholamine‐secreting neuroendocrine tumors that originate from the chromaffin cells of the embryonic neural crest and can be found in the abdomen (most frequently), thorax, pelvis, and neck [1]. PPGLs can be further classified into two subgroups: parasympathetic PPGLs, which are typically nonfunctional, and sympathetic PPGLs, which actively synthesize catecholamines [2].

The incidence of PPGLs is estimated to range from 2 to 8 cases per million people annually, with a prevalence of about 1 in 2500 to 1 in 6500 individuals. These tumors most commonly manifest between the third and fifth decades of life [3]. Paragangliomas most commonly arise in the head and neck region and along the retroperitoneal sympathetic chain. Among extra‐adrenal abdominal paragangliomas, the retroperitoneum is the most frequent location (66.7%), followed by the urinary bladder (19.0%), para‐aortic region (11.9%), and pelvic cavity (2.4%) [4]. Among these, PPGLs occurring in the porta hepatis are exceedingly rare, and to the best of our knowledge, only 15 cases of porta hepatis PPGL have been reported in the literature to date (Table S1).

Key clinical findings suggestive of a PPGL diagnosis include the presence of catecholamine effects, such as hypertension, headaches, sweating, palpitations, and tremors or local manifestations related to the tumor′s growth, such as abdominal pain or discomfort, a palpable mass, or, in some cases, signs of compression affecting nearby structures such as the bile ducts or blood vessels [5].

This case report presents an elderly patient with a rare porta hepatis PPGL with minimal symptoms, highlighting the diagnostic challenges of these rare tumors. The report seeks to enhance understanding of the clinical presentation and diagnostic complexities of porta hepatis PPGLs, which may help clinicians achieve earlier recognition and more accurate diagnoses in similarly rare cases.

## 2. Case Presentation

A 64‐year‐old Iranian woman presented with a 1‐month history of nonspecific abdominal pain. The patient reported episodes of nausea, vomiting, and anorexia. She denied significant weight loss, jaundice, or changes in bowel habits. Her medical history was notable for a cholecystectomy performed 5 years prior. Social history was noncontributory and no relevant family history of cardiovascular, cancer, or endocrine disorders was reported. Notably, there was no history of palpitations, hypertension, hyperhidrosis or tachycardia.

On physical examination, the patient appeared well and was hemodynamically stable. The patient′s vital signs were within normal limits. Cardiovascular and abdominal examinations were unremarkable and there was no tenderness, guarding, palpable masses, or organomegaly.

Routine blood tests, including a complete blood count and metabolic panel, were within normal limits. Aspartate aminotransferase, alanine transaminase, and alkaline phosphatase were 15, 20, and 290 IU/L, respectively. Carbohydrate Antigen 19‐9 (CA 19‐9) and carcinoembryonic antigen were also negative.

Abdominopelvic ultrasonography showed a mass in the porta hepatis region, which was followed up by a contrast‐enhanced CT scan of the abdomen and pelvis, which revealed a well‐defined, isodense mass lesion measuring 3.4 × 2.9 × 2.5 cm without calcification. The mass was located adjacent to the porta hepatis, anterior to the inferior vena cava (IVC), lateral to the crus of the right hemidiaphragm, and posterior to the distal body of the stomach. It demonstrated heterogeneous arterial phase hyperenhancement followed by progressive contrast enhancement during the portal venous phase. Mass effect on IVC with anterior fat plane obliteration without obvious intraluminal invasion was noted. There was also a lymph node lateral to the mass measuring 1.0 × 0.8 cm. No evidence of mass effect on the common bile duct and other adjacent organs was seen. No evidence of communication of the mass to the pancreas and stomach was seen.

Given the nonspecific symptoms and lack of classical adrenergic symptoms, and also the location of the mass, PPGL was not included in the initial differential diagnosis and a core needle biopsy was performed in another institution. The core needle biopsy was completed without immediate hemodynamic complications. The preliminary histopathology report described a bland spindle cell proliferation favoring a benign mesenchymal neoplasm; however, a specific mesenchymal entity was not definitively specified, and the biopsy report did not clarify if immunohistochemical (IHC) markers were performed at that stage.

The patient underwent surgery, and the tumor was confirmed to be closely positioned near the IVC, consistent with the imaging findings. During the operation, the patient had an episode of increased blood pressure that was managed by the anesthesia team. The tumor was successfully removed, and no local invasion was found during the exploration of the abdominal cavity. The specimen was sent to the pathology department for histopathological examination.

Gross examination of the fresh specimen, performed prior to formalin fixation, revealed a well‐circumscribed, grayish, firm mass measuring 3.6 × 3.0 × 2.5 cm. On cut sections, the mass was partially encapsulated with a brownish‐red hemorrhagic surface and scattered cream‐colored areas. Light microscopy of the entire examined tissue with H&E stain showed round to polygonal epithelioid cells with eosinophilic granular cytoplasm arranged in nests (zellballen pattern) separated by prominent fibrovascular stroma (Figure 1). The epithelioid cells showed mild nuclear atypia without evidence of mitotic figures, necrosis, or multinucleated giant cells. On IHC evaluation of the resected specimen, the neoplastic cells demonstrated strong, diffuse cytoplasmic positivity for Synaptophysin (SYN) (Figure 2) and Chromogranin A (CGA) (Figure 3). S100 staining highlighted a distinct population of peripheral sustentacular cells flanking the cell nests (Figure 4). The Ki‐67 proliferation index was formally quantified and measured at < 1% (Figure 5). The tumor cells were entirely negative for cytokeratin AE1/AE3 (CK AE1/AE3) (Figure 6), CEA, and CD34, though CD34 highlighted a rich background network of delicate capillaries (Figure 7).Because of institutional resource constraints at the time of evaluation, insulinoma‐associated protein 1 (INSM1) staining was unavailable and could not be performed. Reticulin stain highlighted the nesting pattern (Figure 8). Because of institutional resource constraints, SDHB immunohistochemistry—a critical prognostic surrogate marker for SDHx germline mutations and higher metastatic risk—could not be performed. Finally, a diagnosis of PPGL was made for the patient, with no evidence of capsular invasion.

**Figure 1:**
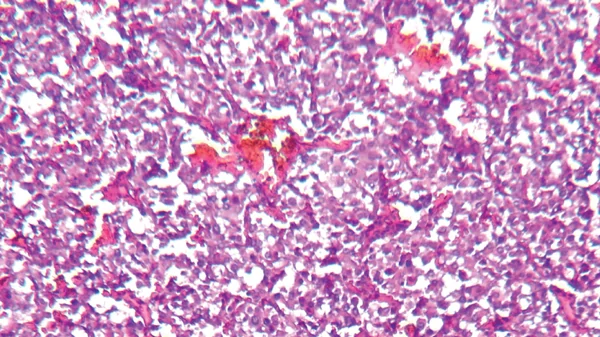
Epithelioid cells with eosinophilic granular cytoplasm arranged in nests (zellballen pattern), separated by prominent fibrovascular stroma (H&E staining, ×400).

**Figure 2:**
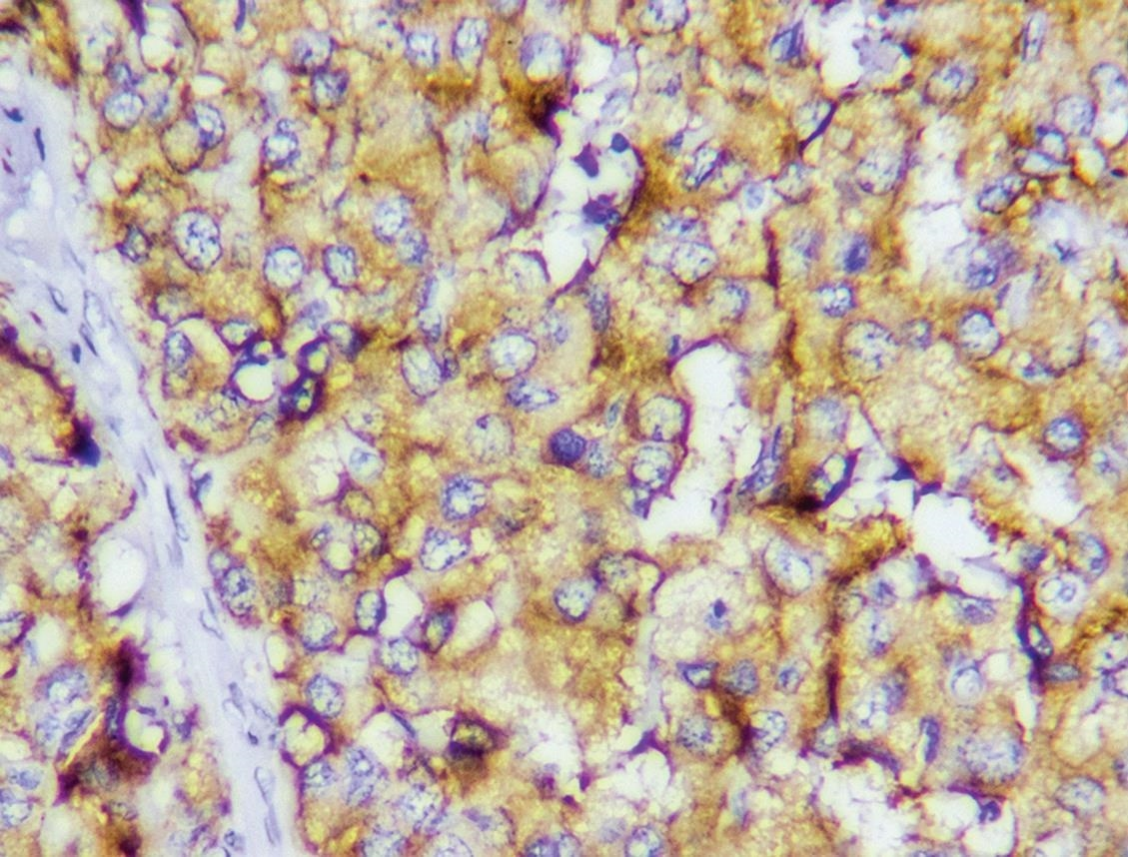
Positive cytoplasmic synaptophysin in tumor cells (IHC stain, ×400).

**Figure 3:**
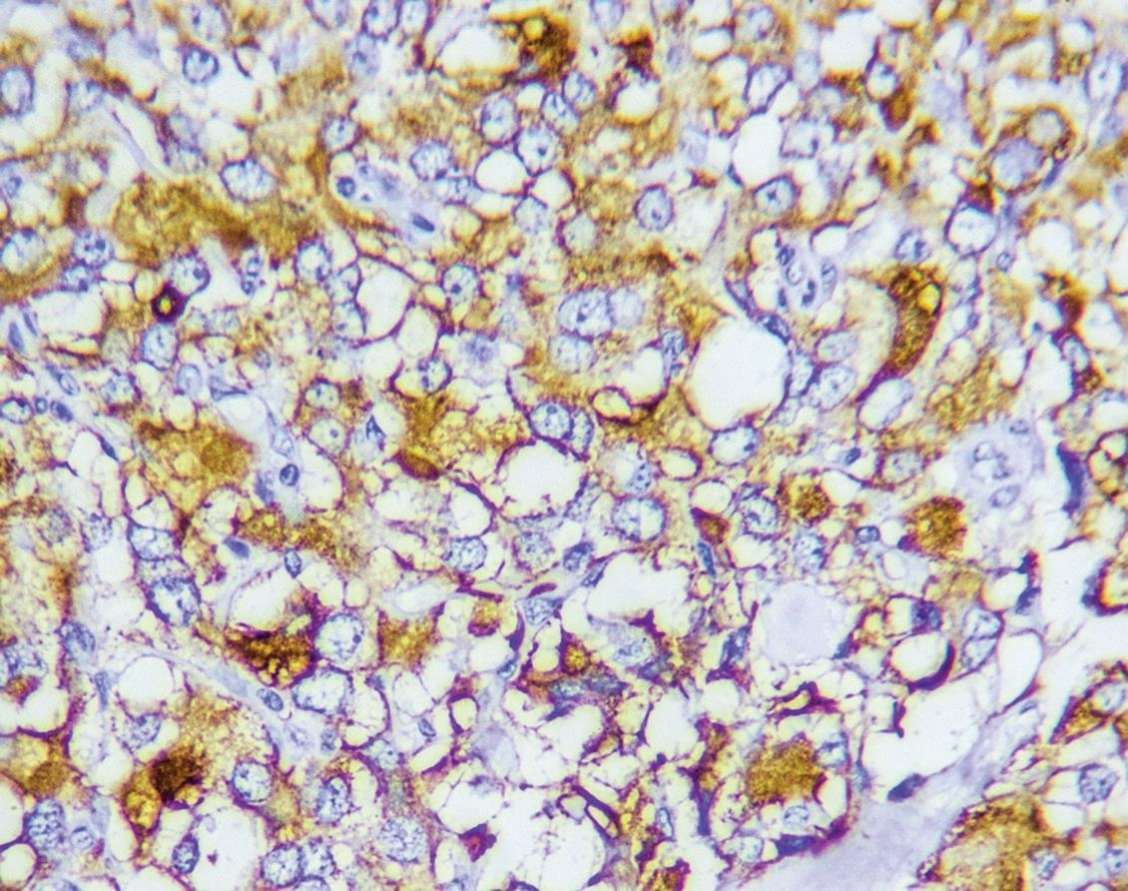
Positive chromogranin A (IHC stain, ×400).

**Figure 4:**
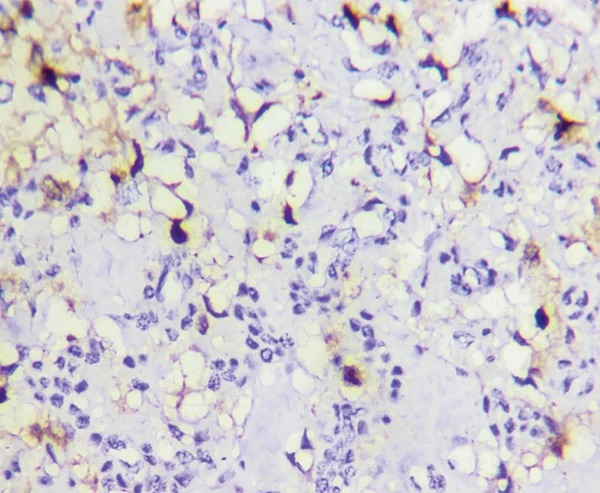
S100 marker accentuates sustentacular cells with intense nuclear and cytoplasmic stain (IHC stain, ×400).

**Figure 5:**
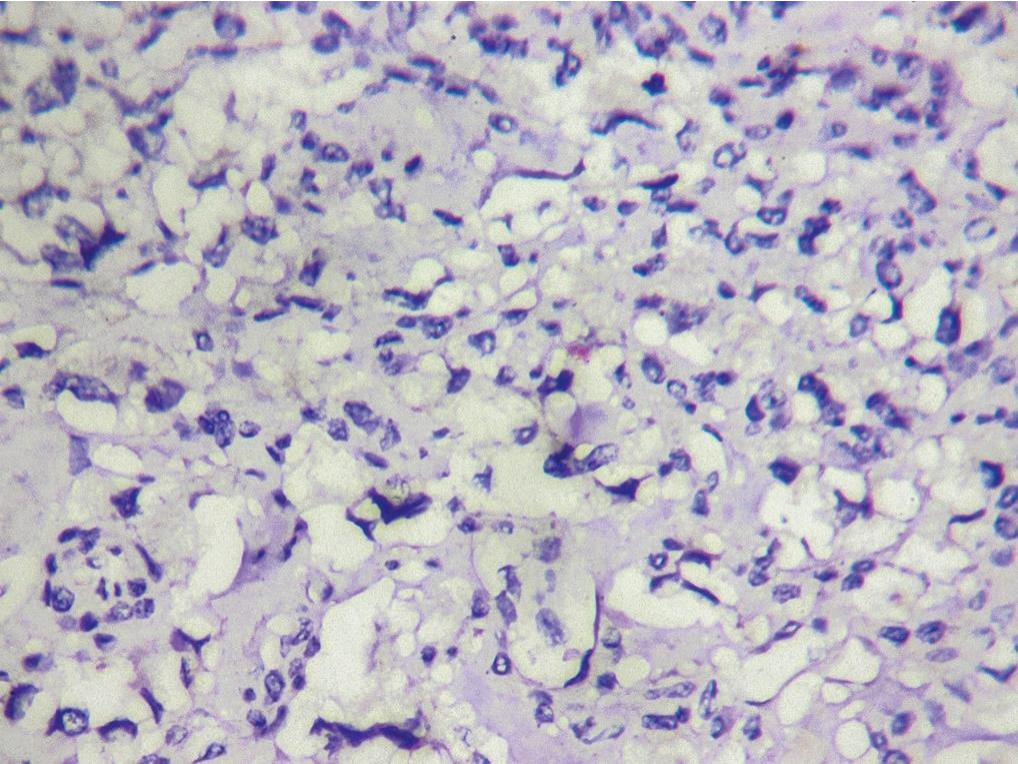
Ki67 proliferative index < 1% (IHC stain, ×400).

**Figure 6:**
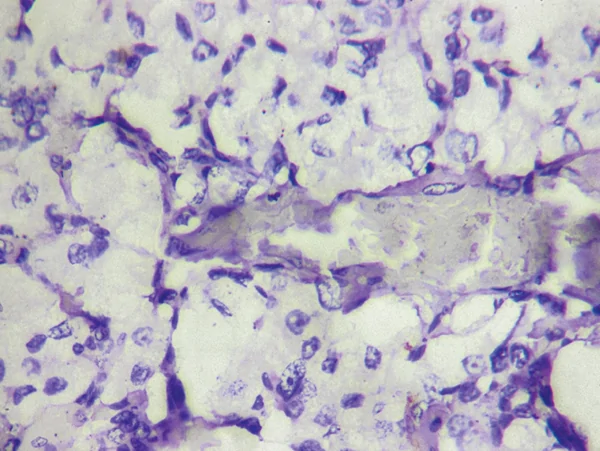
Negative CKAE1/AE3 (IHC stain, ×400).

**Figure 7:**
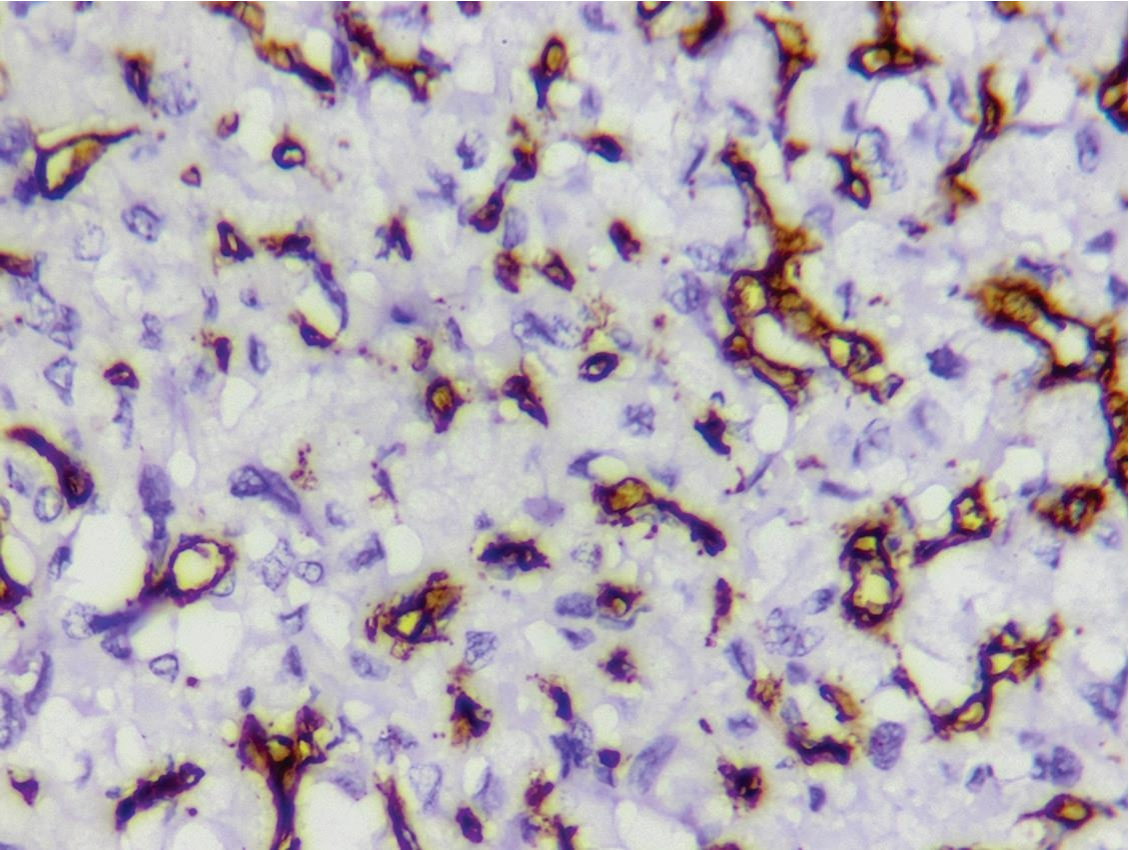
CD34 showed rich vascularity in paraganglioma, but was negative in the tumor cells (IHC stain, ×400).

**Figure 8:**
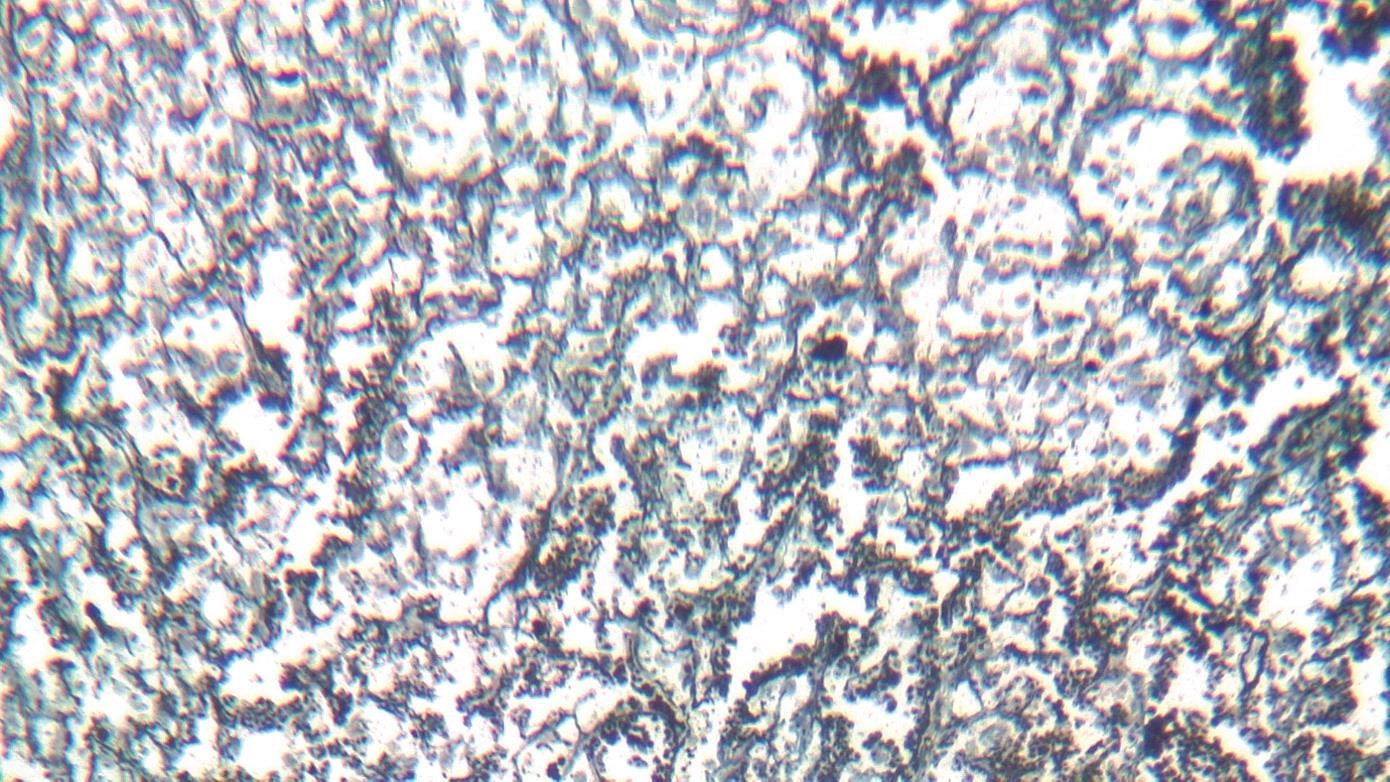
Hepatic paraganglioma, reticulin stain highlights nesting pattern (reticulin stain, ×250).

The patient′s postoperative recovery was stable, and she was discharged 5 days following surgery. To address the risk of an unblockaded intraoperative hypertensive event, targeted postoperative biochemical testing was performed: both plasma free metanephrines and urinary fractionated metanephrines were measured and found to be completely within normal physiologic limits. She has been confirmed to remain completely asymptomatic with no signs of disease recurrence during the two‐year evaluation window so far.

## 3. Discussion

Although porta hepatis PPGLs are rare, they should remain part of the differential diagnosis for abdominal masses, particularly when other more common diagnoses are ruled out. Functional PPGLs typically present with catecholamine‐related symptoms such as episodic headaches, sweating, palpitations, and paroxysmal hypertension [6, 7]. These patients are at a heightened risk of life‐threatening cardiovascular complications [8]. However, 10%–15% of PPGLs are nonfunctioning, and in another 10%, hormonal activity is not clinically evident [9]. Nonfunctional tumors remain clinically silent until mass‐effect complications emerge; previously reported cases have documented impairment of nearby systems, such as the hepatic parenchyma or bile ducts, resulting in obstructive jaundice [10–13]. When primary extra‐adrenal paragangliomas emerge in atypical or ultra‐rare anatomical boundaries, they consistently mirror alternate local primary lesions. Beyond the classic retroperitoneum and head and neck areas, primary tumors have been documented within the urinary bladder, mediastinum, trachea, deep pelvic soft tissues, and the organ of Zuckerkandl [14]. When presenting in these unusual locations—particularly when lacking classical symptoms of catecholamine hypersecretion—preoperative clinical differentiation becomes exceptionally difficult. Some abdominal PPGLs are found incidentally by imaging studies and some may mimic more common neoplasms, such as hepatocellular carcinoma (HCC) and gastrointestinal stromal tumor (GIST). The imaging features of abdominal PPGLs, such as their appearance on CT, MRI, or ultrasound, are often not specific enough to reliably differentiate them from HCC or other more common abdominal masses [15–17]. Limited core biopsy tissue can capture only localized fragments of bland architecture or vascularity, easily leading a diagnostic team to mistake the tumor for other hypervascular mesenchymal, stromal, or epithelial mimics, such as GISTs, schwannomas, leiomyomas, metastatic carcinomas, or primary neuroendocrine tumors. The present case had limited evidence to help us reach a diagnosis of porta hepatis PPGL. The only symptom the patient had was abdominal pain, and preoperatively, there was no history of catecholamine release symptoms.

Histopathological examination remains the gold standard for definitive diagnosis of PPGL; however, performing a core needle biopsy on an unsuspected, unblockaded PPGL is discouraged due to an almost 15% chance of serious complications, including potentially life‐threatening adrenergic crisis due to mechanical tumor disruption [18]. In the present case, a low initial clinical index of suspicion for a PPGL prompted a core needle biopsy, which erroneously characterized the mass as a benign mesenchymal neoplasm. Guided by this preliminary diagnostic impression, the multidisciplinary team proceeded with definitive surgical resection of the tumor. PPGL was confirmed postsurgery through immunohistochemical analysis.

The classic histological pattern seen in PPGLs is the “zellballen” structure, which consists of nests of tumor cells separated by delicate fibrovascular stroma. Common neuroendocrine markers used for PPGL identification include CGA and SYN. Tyrosine hydroxylase is a more specific marker. Sustentacular cells surrounding the zellballen can be identified using S100 staining. The absence of keratin can distinguish PPGLs from other neuroendocrine tumors [19]. In cases of suspicion of PPGLs, biochemical tests to assess levels of metanephrines are recommended. Measuring either urinary or plasma metanephrines is considered an accurate method for detecting the presence of these tumors. However, it is important to note that not all PPGLs raise the metanephrines levels, and this can be misleading [20]. Some recent studies suggest that 30%–40% of PPGL originate from hereditary disease, and some germline mutations in the succinate dehydrogenase genes are responsible for that [21, 22]. Given that extra‐adrenal paragangliomas are frequently associated with germline mutations (up to 40%–50%), offering germline genetic testing is a key aspect of current clinical management standards. In our case, this evaluation was offered to the patient; however, she declined the testing due to the high costs.

Surgical resection remains the primary treatment modality. Patients with functional PPGLs need preoperative blockade to minimize surgical complications. Additionally, preparation should include a high‐sodium diet and increased fluid intake to lower the risk of postoperative hypotension [23]. However, Liao et al. reported that from 11 hepatic PPGL cases, only two cases received a correct diagnosis before surgery, and most patients were at risk of catecholamine‐release complications like hypertension crisis during the surgery same as our case, underscoring the importance of preoperative diagnosis [2].

Historically, 10%–20% of PPGLs were classified as malignant; however, the 2022 WHO Classification of Endocrine and Neuroendocrine Tumors establishes that all PPGLs possess variable metastatic potential, rendering the term “benign” obsolete in this context [24]. To better characterize biological behavior, histopathologic scoring systems such as the Pheochromocytoma of the Adrenal Gland Scaled Score (PASS) and the Grading System for Adrenal Pheochromocytoma and Paraganglioma (GAPP) are frequently discussed in literature. Although the PASS system remains a common standard for adrenal tumors, its clinical utility and prognostic accuracy are significantly limited when applied to extra‐adrenal paragangliomas. Therefore, we structured our postoperative evaluation primarily around the GAPP scoring framework. Histologically, our patient′s tumor showed a classical zellballen pattern, moderate cellular density, an absolute absence of tumor necrosis, no capsular or vascular invasion, and a Ki‐67 index of < 1%. Under these strict parameters, the mass achieved a GAPP score of 0, successfully stratifying it as a well‐differentiated paraganglioma carrying a very low predicted metastatic potential [25–27]. However, reliance on the GAPP score alone is problematic in light of recent findings. Newer prognostic models, such as the composite pheochromocytoma/paraganglioma prognostic score (COPPS) and the age, size, extra‐adrenal location, secretory type (ASES) score, offer superior predictive performance and higher C‐indices compared with GAPP [28]. When applied to our patient, the ASES score remains strictly below the high‐risk cutoff, as well as COPPS score. Although SDHB immunohistochemistry was unavailable (which contributes a maximum of 1 point if lost), the highest potential COPPS score is 1, remaining well below the ≥ 3 threshold for high metastatic risk. Consequently, these superior scoring systems independently corroborate the low‐risk stratification for this patient [28].

Current international guidelines recommend somatostatin receptor‐based functional imaging, specifically 68Ga‐DOTATATE PET/CT, as the first‐line imaging modality for the comprehensive staging and long‐term surveillance of extra‐adrenal paragangliomas due to its superior sensitivity for detecting multifocal or metastatic disease [29]. Because of institutional resource constraints, this modality was unavailable at our center. Consequently, the patient′s surveillance has been meticulously managed using serial biochemical evaluations and standard imaging, which have confirmed a stable, disease‐free course over the 2‐year follow‐up period.

## Funding

No funding was received for this manuscript.

## Consent

Written informed consent was obtained from the patient for the publication of this case report and any accompanying deidentified clinical, radiological, and histopathological images.

## Conflicts of Interest

The authors declare no conflicts of interest.

## Supporting Information

Additional supporting information can be found online in the Supporting Information section.

## Supporting information


**Supporting Information 1** Table S1: Literature review of reported cases of porta hepatis region paragangliomas (excluding gallbladder paragangliomas).

**Supporting Information 2** File S1: CARE checklist.

## Data Availability

The data that support the findings of this study are available on request from the corresponding author. The data are not publicly available due to privacy or ethical restrictions.
