# Supplementary material for: Porta Hepatis Paraganglioma: A Rare Case Report With Diagnostic Pitfalls
Source: Case Rep Pathol. 2026 Oct 21;2026:4237665. doi: 10.1155/crip/4237665 (PMC13687654; doi:10.1155/crip/4237665)
Supplement: Supplementary file 1 [file CRIP-2026-4237665-s001.docx]

Supplement Table 1: Reported cases of Porta hepatis region paragangliomas (excluding gallbladder paragangliomas)

| # | Reference | Age | Sex (M= male; F= female) | Location | Presentation | Extra |
| --- | --- | --- | --- | --- | --- | --- |
| 1 | (Sarma et al., 1980) | 37 | M | Hepatic duct | Obstructive jaundice |  |
| 2 | (Hitanant et al., 1984) | 59 | M | Common bile duct | Obstructive jaundice | *Full-text could not be retrieved* |
|  | (Davis, 1987) | 36 | F | Porta hepatis | Acute abdominal pain (RUQ) |  |
| 3 | (Sandhu et al., 2000) | young | F | Extrahepatic biliary tract | Obstructive jaundice | *Full-text could not be retrieved* |
| 4 | (Caceres et al., 2001) | 28 | F | Bile duct | Abdominal pain (RLQ) |  |
| 5 | (Prakash et al., 2013) | 32 | F | Bile duct | Obstructive jaundice & weight loss |  |
| 6 | (Jawad et al., 2017) | 26 | F | Porta hepatis | Acute left-sided chest pain, palpitations and dizziness |  |
| 7 | (Yadav et al., 2018) | 48 | F | Portocaval space | Diffuse dull upper abdominal pain |  |
| 8 | (AlMarzooqi et al., 2018) | N/A | F | Cystic duct | Acute abdominal pain (RUQ) |  |
| 9 | (Khare et al., 2018) | 35 | F | Porta hepatis | Abdominal pain (lower) & weight loss |  |
| 10 | (Rasquin et al., 2018) | 32 | M | Porta hepatis | Intermittent abdominal pain & episodic axillary hyperhidrosis | liver mass during surgical exploration for an acute appendicitis |
| 11 | (Ghajar et al., 2022) | 24 | M | Porta hepatis | Headaches, hypertension, hyperhidrosis |  |
| 12 | (Pirzada et al., 2022) | 55 | F | Common bile duct | Jaundice and RLQ discomfort |  |
| 13 | (Azad et al., 2023) | 76 | M | Porta hepatis | Asymptomatic; Incidental finding in CT for lung nodule surveillance |  |
| 14 | (Atogwe et al., 2025) | 29 | M | Porta hepatis | Asymptomatic; elevated liver enzymes |  |
| 15 | (Salameh et al., 2025) | 16 | F | Retroperitoneum + Porta hepatis | Acute chest pain, headache, dyspnea, and palpitations |  |
| 16 | *Our case* | 64 | F | Porta hepatis | Abdominal pain (RLQ) |  |

**References:**

AlMarzooqi, R., AlJaberi, L., Rosenblatt, S., Plesec, T., & Berber, E. (2018). A rare case of paraganglioma of the cystic duct. *Int J Surg Case Rep*, *52*, 16-19. <https://doi.org/10.1016/j.ijscr.2018.09.041>

Atogwe, I. D., Bathobakae, L., Deepali, F., Lagarde Mussa, M., & Shoyele, O. (2025). Periportal Paraganglioma: A Rare Cause of Obstructive Jaundice and Gastroesophageal Varices. *ACG Case Reports Journal*, *12*(6), e01742. <https://doi.org/10.14309/crj.0000000000001742>

Azad, F., Carlson, A. M., Miranda, C. J., Patel, P., & Ayaz, M. (2023). S3705 Porta Hepatis Paraganglioma in a Patient with Lung Adenocarcinoma. *Official journal of the American College of Gastroenterology| ACG*, *118*(10S), S2396.

Caceres, M., Mosquera, L. F., Shih, J. A., & O'Leary, J. P. (2001). Paraganglioma of the bile duct. *South Med J*, *94*(5), 515-518.

Davis, J. A. (1987). Ultrasonic Demonstration of Paraganglioma. *Journal of Diagnostic Medical Sonography*, *3*, 289 - 292.

Ghajar, D., Khana, F., Zakkor, M. D., AlHussein, H., & Kayyali, A. (2022). Portocaval paraganglioma: A second case report. *Annals of Medicine and Surgery*, *80*. <https://doi.org/10.1016/j.amsu.2022.104236>

Hitanant, S., Sriumpai, S., Na-songkla, S., Pichyangkula, C., Sindhavananda, K., & Viranuvatti, V. (1984). Paraganglioma of the common hepatic duct. *Am J Gastroenterol*, *79*(6), 485-488.

Jawad, Z. A. R., Fajardo-Puerta, A. B., Lefroy, D., Todd, J., Lim, P. B., & Jiao, L. R. (2017). Complete laparoscopic excision of a giant retroperitoneal paraganglioma. *The Annals of The Royal College of Surgeons of England*, *99*(5), e148-e150. <https://doi.org/10.1308/rcsann.2017.0056>

Khare, M., Gupta, M. K., Sharma, U. B., & Garg, K. M. (2018). Paraganglioma with a very rare presentation. *Indian Journal of Pathology and Microbiology*, *61*(3), 404-406. <https://doi.org/10.4103/ijpm.Ijpm_334_17>

Pirzada, M. A., Khan, M. R., Begum, S., & Minhas, K. (2022). Paraganglioma of common bile duct: A case report. *J Pak Med Assoc*, *72*(5), 983-985. <https://doi.org/10.47391/jpma.4286>

Prakash, K., Kamalesh, N. P., & Mathew, P. (2013). Malignant paraganglioma of the bile duct. *Trop Gastroenterol*, *34*(3), 180-182.

Rasquin, L., Prater, J., Mayrin, J., & Minimo, C. (2018). Simultaneous Pheochromocytoma, Paraganglioma, and Papillary Thyroid Carcinoma without Known Mutation. *Case Reports in Endocrinology*, *2018*(1), 6358485. <https://doi.org/https://doi.org/10.1155/2018/6358485>

Salameh, M., Swizer, K., Patel, A. P., & Petrescu, M. (2025). Acute Cardiogenic Shock Requiring Extracorporeal Membrane Oxygenation Secondary to Catecholamine-Secreting Paraganglioma: A Case Report. *Cureus*, *17*(2), e79696. <https://doi.org/10.7759/cureus.79696>

Sandhu, B. S., Kumar, N., Sachdeva, A. K., Negi, S. S., Sridhar, S., Malhotra, V., Lamba, G. S., & Puri, A. S. (2000). Paraganglionoma of extrahepatic biliary tract causing obstructive jaundice. *Indian J Gastroenterol*, *19*(3), 141-142.

Sarma, D. P., Rodriguez, F. H., Jr., & Hoffmann, E. O. (1980). Paraganglioma of the hepatic duct. *South Med J*, *73*(12), 1677-1678. <https://doi.org/10.1097/00007611-198012000-00050>

Yadav, K., Taneja, S., Dang, R., Menon, S., Kapoor, G., Patil, A., & Banwari, A. (2018). Portocaval Paraganglioma : First Case Report and Review of Literature. *Journal of Gastrointestinal Cancer*, *49*(3), 355-357. <https://doi.org/10.1007/s12029-017-9917-x>
