# Supplementary material for: Porta Hepatis Paraganglioma: A Rare Case Report With Diagnostic Pitfalls
Source: Case Rep Pathol. 2026 Oct 21;2026:4237665. doi: 10.1155/crip/4237665 (PMC13687654; doi:10.1155/crip/4237665)
Supplement: Supplementary file 2 [file CRIP-2026-4237665-s002.docx]

# CARE Checklist

| Topic / Item | CARE Checklist Item Description | Specific Information from the Case Report | Exact Quote from the Manuscript |
| --- | --- | --- | --- |
| Title    *Item 1* | The diagnosis or intervention of primary focus followed by the words "case report". | **Porta Hepatis Paraganglioma** mimicking a mesenchymal neoplasm, presented as a **Case Report**. | *"Porta Hepatis Paraganglioma Mimicking Mesenchymal Neoplasm on Core Needle Biopsy: A Case Report"* |
| Keywords    *Item 2* | 2 to 5 keywords that identify diagnoses or interventions, including "case report". | Provided 5 high-yield keywords. | *"Keywords: Paraganglioma; Porta hepatis; Biopsy, Large-Core Needle; Diagnostic Errors; Case Report."* |
| Abstract    *Item 3a* | **Introduction:** What is unique about this case and what does it add to the literature? | Ultra-rare localization of a paraganglioma at the porta hepatis mimicking mesenchymal/hepatic tumors. | *"While the retroperitoneum is the most common site, localization to the porta hepatis is exceedingly uncommon... Their clinical and radiological presentation often overlaps with more common hepatic or mesenchymal tumors, making preoperative diagnosis highly challenging."* |
| Abstract    *Item 3b* | **Main symptoms and/or important clinical findings:** | A 64-year-old female presenting with 1 month of vague abdominal symptoms; CT showing a porta hepatis mass compressing the IVC; initial core biopsy misdiagnosed it. | *"We report the case of a 64-year-old woman presenting with vague abdominal pain, nausea, and anorexia of one month’s duration... imaging revealed a well-defined, enhancing mass... A core needle biopsy suggested a benign mesenchymal neoplasm."* |
| Abstract    *Item 3c* | **The main diagnoses, therapeutic interventions, and outcomes:** | Definitive diagnosis of paraganglioma confirmed by surgical pathology; management via resection; disease-free status at 2 years. | *"The patient underwent surgical resection, complicated by paroxysmal intraoperative hypertension. Subsequent histopathological examination... confirming the diagnosis of paraganglioma. The patient remained stable postoperatively and showed no recurrence at two-year follow-up."* |
| Abstract    *Item 3d* | **Conclusion—What is the main "take-away" lesson?** | Clinical/pathological pitfalls of limited biopsies in silent PGLs; critical need to anticipate intraoperative catecholamine crises. | *"This case illustrates the diagnostic pitfalls associated with porta hepatis paragangliomas... Awareness of this rare entity is crucial... to anticipate intraoperative catecholamine-related hemodynamic instability, even in the absence of overt biochemical symptoms."* |
| Introduction    *Item 4* | One or two paragraphs summarizing why this case is unique (with references). | Outlines classic epidemiology/locations of PPGLs, notes the exceptional rarity at the porta hepatis, and outlines the educational objectives of reporting minimal symptoms. | *"Among these, PGLs occurring in the porta hepatis are exceedingly rare... This case report investigates an elderly patient with a rare porta hepatis PGL with minimal symptoms... seeks to enhance understanding of the clinical presentation and diagnostic complexities"* |
| Patient Information    *Item 5a* | De-identified patient-specific demographic information. | 64-year-old Iranian female. | *"A 64-year-old Iranian woman..."* |
| Patient Information    *Item 5b* | Primary concerns and symptoms of the patient. | 1-month history of abdominal pain, nausea, vomiting, and anorexia. | *"...presented with a one-month history of non-specific abdominal pain. The patient reported episodes of nausea, vomiting, and anorexia."* |
| Patient Information    *Item 5c* | Medical, family, and psychosocial history including genetic details. | Medical history: Cholecystectomy 5 years ago. Negative family history of endocrine/cardiovascular disease. Absence of adrenergic symptoms. | *"Her medical history was notable for a cholecystectomy performed five years prior... no relevant family history of cardiovascular, cancer, or endocrine disorders... there was no history of palpitations, hypertension, hyperhidrosis or tachycardia."* |
| Patient Information    *Item 5d* | Relevant past interventions with outcomes. | Cholecystectomy 5 years prior, completed without reported relevant long-term sequelae. | *"Her medical history was notable for a cholecystectomy performed five years prior."* |
| Clinical Findings    *Item 6* | Significant physical examination (PE) and important clinical findings. | Patient appeared well and was completely hemodynamically stable; abdominal exam was entirely unrevealing. | *"On physical examination, the patient appeared well and was hemodynamically stable... abdominal examinations were unremarkable and there was no tenderness, guarding, palpable masses, or organomegaly."* |
| Timeline    *Item 7* | Historical and current information from this episode organized as a timeline. | Summarized chronological milestones spanning symptom onset, radiological workup, core needle biopsy, operative resection, postoperative testing, and 2-year surveillance. | *(See the compiled chronological timeline below this checklist table for direct implementation into your manuscript template).* |
| Diagnostic Assessment    *Item 8a* | Diagnostic testing (PE, laboratory testing, imaging, surveys). | • Normal routine labs/liver enzymes/tumor markers .    • US & contrast CT showing a 3.4×2.9×2.5 cm arterial hyperenhancing porta hepatis mass compressing the IVC .    • Post-op metanephrines. | *"...CT scan... revealed a well-defined, isodense mass lesion measuring 3.4 × 2.9 × 2.5 cm... demonstrated heterogeneous arterial phase hyperenhancement followed by progressive contrast enhancement... Mass effect on IVC..."* |
| Diagnostic Assessment    *Item 8b* | Diagnostic challenges (such as access, financial, or cultural; false-negative biopsy). | Misleading preoperative core biopsy showing bland spindle cells; complete absence of classical adrenergic symptoms; lack of access to INSM1 stain. | *"The preliminary histopathology report described a bland spindle cell proliferation favoring a benign mesenchymal neoplasm... Due to institutional resource constraints... insulinoma-associated protein 1 (INSM1) staining was unavailable..."* |
| Diagnostic Assessment    *Item 8c* | Final diagnosis (including other diagnoses considered). | Final diagnosis: Paraganglioma (PGL) of the porta hepatis. Initial differentials: benign mesenchymal neoplasm, HCC, GIST. | *"...Finally, a diagnosis of PGL was made for the patient, with no evidence of capsular invasion."* |
| Diagnostic Assessment    *Item 8d* | Prognosis (such as staging in oncology) where applicable. | Low metastatic potential calculated using a formal GAPP score of 0. | *"...the mass achieved a GAPP score of 0, successfully stratifying it as a well-differentiated paraganglioma carrying a very low predicted metastatic potential"* |
| Therapeutic Intervention    *Item 9a* | Types of therapeutic intervention (such as pharmacologic, surgical, preventive). | Complete exploratory laparotomy and surgical tumor removal. | *"The patient underwent surgery... The tumor was successfully removed, and no local invasion was found during the exploration of the abdominal cavity."* |
| Therapeutic Intervention    *Item 9b* | Administration of therapeutic intervention (such as dosage, strength, duration). | Performed surgically under general anesthesia; required immediate anesthetic management for an intraoperative hypertensive crisis. | *"During the operation, the patient had an episode of increased blood pressure that was managed by the anesthesia team."* |
| Therapeutic Intervention    *Item 9c* | Changes in therapeutic intervention (with rationale). | No procedural changes; complete, uncomplicated gross surgical excision was successfully finalized. | *"The tumor was successfully removed..."* |
| Follow-up and Outcomes    *Item 10a* | Clinician and patient-assessed outcomes (if available). | Completely stable clinical course with smooth postoperative recovery and early discharge. | *"The patient’s post-operative recovery was stable, and she was discharged five days following surgery."* |
| Follow-up and Outcomes    *Item 10b* | Important follow-up diagnostic and other test results. | Post-op plasma free metanephrines and urinary fractionated metanephrines were entirely normal. | *"...targeted post-operative biochemical testing was performed: both plasma free metanephrines and urinary fractionated metanephrines were measured and found to be completely within normal physiologic limits."* |
| Follow-up and Outcomes    *Item 10c* | Intervention adherence and tolerability (How was this assessed?). | Excellent surgical tolerability confirmed through clinical evaluations across a 2-year surveillance period. | *"She has been confirmed to remain completely asymptomatic with no signs of disease recurrence during two-year evaluation window so far."* |
| Follow-up and Outcomes    *Item 10d* | Adverse and unanticipated events. | Unanticipated, dangerous paroxysmal intraoperative hypertensive event triggered by mechanical manipulation of an un-blockaded functional tumor. | *"During the operation, the patient had an episode of increased blood pressure... performing a core needle biopsy [or surgery] on an unsuspected, un-blockaded catecholamine-secreting tumor carries an immense risk of triggering a catastrophic... adrenergic crisis"* |
| Discussion    *Item 11a* | A scientific discussion of the strengths AND limitations associated with this case report. | • **Strengths:** 2-year disease-free tracking , extensive IHC panels , GAPP scoring .    • **Limitations:** Initial biopsy error , lack of pre-op blockade , unavailable INSM1. | "...emphasizes the potential for misdiagnosis based on pathological findings alone ... Due to institutional resource constraints... INSM1 staining was unavailable ... structured our post-operative evaluation primarily around the GAPP scoring framework." |
|  | **Discussion**    *Item 11b* | Discussion of the relevant medical literature with references. | Extensively contextualizes abdominal extra-adrenal PGL sites , silent/nonfunctional clinical presentations , radiologic/biopsy pitfalls mimicking HCC/GIST , biopsy crisis risks , and systemic review of 11 earlier hepatic cases. |
| Discussion    *Item 11c* | The scientific rationale for any conclusions. | Confirms that microscopic Zellballen nest configurations combined with positive neuroendocrine stains (SYN/CGA), outer S100+ scaffolding, and cytokeratin negativity provide a reliable diagnostic standard. | *"Definitive diagnosis relies on histopathology and immunohistochemistry... Zellballen structure... Common neuroendocrine markers... CGA and SYN... S100 staining... absence of keratin can distinguish PGLs..."* |
| Discussion    *Item 11d* | The primary "take-away" lessons of this case report in a one-paragraph conclusion. | Clinicians and pathologists must keep extra-adrenal PGL in mind for atypical masses to anticipate intraoperative crises, even when baseline adrenergic parameters are silent. | *"Awareness of this rare entity is crucial for clinicians and pathologists to avoid misdiagnosis and to anticipate intraoperative catecholamine-related hemodynamic instability, even in the absence of overt biochemical symptoms."* |
| Patient Perspective    *Item 12* | The patient should share their perspective on the treatment received. | Not explicitly detailed in the manuscript text, though the patient reports a high quality of life and a smooth, asymptomatic recovery. | *"...patient has remained asymptomatic without any evidence of recurrence."* |
| Informed Consent    *Item 13* | Did the patient provide informed consent? Please provide if requested. | Yes. Obtained complete written informed consent for publication and de-identified figures. | *"Written informed consent was obtained from the patient for the publication of this case report and any accompanying de-identified clinical, radiological, and histopathological images."* |
